# Phase-sensitive black-blood coronary vessel wall imaging

**DOI:** 10.1186/1532-429X-11-S1-O11

**Published:** 2009-01-28

**Authors:** Khaled Z Abd-Elmoniem, Matthias Stuber

**Affiliations:** Department of Radiology, School of Medicine, Johns Hopkins University, Baltimore, MD USA

**Keywords:** Right Coronary Artery, Coronary Artery Vessel, Vessel Wall Imaging, Wall Thickness Measurement, Image Data Collection

## Introduction

Black-blood coronary vessel wall imaging is a powerful non-invasive tool for the quantitative assessment of positive arterial remodeling [1]. Although dual-inversion-recovery [2] (DIR) is the gold standard for vessel wall imaging, optimal lumen-vessel wall contrast is sometimes difficult to obtain and the time-window available for imaging is limited due to the competing requirements between TI* (blood signal nulling time) and TD (period of minimal myocardial motion). In addition, atherosclerosis is a spatially heterogeneous disease and therefore imaging at multiple anatomical levels of the coronary circulation is mandatory. However, this requirement of enhanced volumetric coverage typically comes at the expense of increased scanning time. Phase-sensitive IR [3–5] (PS-IR) has shown to be valuable for enhancing tissue-tissue contrast and for making IR imaging less sensitive to TI*. This work extends PS-IR to PS-DIR and combined with spiral-imaging, multi-slice black-blood coronary vessel wall imaging is enabled in a single breath-hold.

## Purpose

To develop, and test a phase-sensitive DIR (PS-DIR) single-breath-hold multi-slice spiral black-blood coronary vessel wall imaging method.

## Methods

### Concept

**Figure 1:**
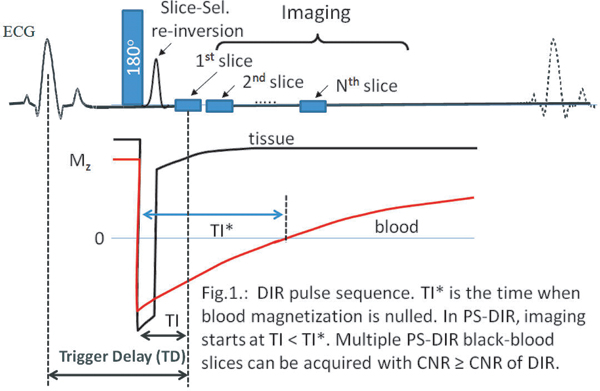
**DIR pulse sequence**. TI* is the time when blood magnetization is nulled. In PS-DIR, imaging starts at TI < TI*. Multiple PS-DIR black-blood slices can be acquired with CNR ≥ CNR of DIR.

After DIR (Fig. 1), the inversion time TI* allows for signal-nulling of the in-flowing blood-pool at the anatomical level of interest. Blood-tissue contrast therefore depends on the accurate determination of TI*. Although the MR signal is complex (magnitude and phase), DIR images only show the magnitude of the signal with a suboptimal blood-tissue contrast if TI does not equal TI*. However, by additionally using the MR signal phase, a signed (positive/negative) black-blood image can be acquired at TI less than TI* and reconstructed with a blood-tissue contrast higher than that obtained at TI*. Simultaneously, competing constraints related to TI* and TD are avoided (Fig. 1). Consequently, single-breath-hold multi-slice black-blood coronary vessel wall imaging is enabled using PS-DIR.

### Reconstruction

**Figure 2:**
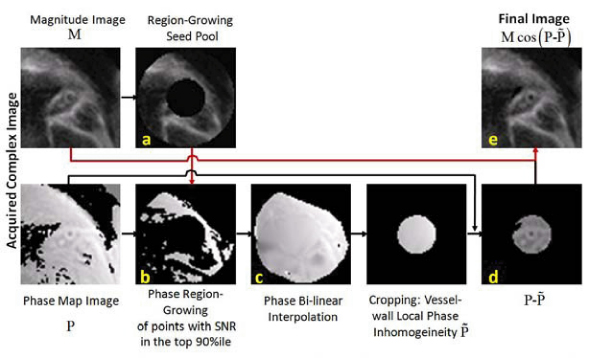
**Summary of the PS-DIR Coronary wall signed-image reconstruction algorithm**. A ring-shaped region-of-interest is selected around the coronary wall (a). Phase points with high magnitude SNR are selected for region-growing (b). Map of local phase inhomogeneity is created using bi-linear interpolation (c). Inhomogeneity is removed from the phase image (d) and a final signed image is calculated (e).

A local region-growing reconstruction algorithm was developed and is summarized in Fig. 2. Pixels with high signal near the cross-sectional coronary artery are selected as seed points. The phase values of these points are used to estimate the local signal phase inhomogeneity which is needed for local signed-magnitude image reconstruction [4].

### Implementation

A single breathhold DIR sequence was implemented (Fig. 1) on a clinical 3 T Philips-Achieva MRI-system. Data were acquired using a segmented k-space spiral acquisition with spectral spatial excitation [6]. Image processing was performed off-line on a personal computer.

## Experiments

Anatomical slices perpendicular to the proximal part of the right coronary artery (RCA) at end-systole were planned similar to a previously published methodology [7]. First, serial single-slice multi-phase PS-DIR images were acquired with incremental TI ranging from 50 ms–500 ms in 15 healthy adult subjects (slice-thickness = 8 mm, FOV = 190 × 190 mm, matrix = 320 × 320, interleaves = 20, acq.window = 18 ms/interleaf). CNR was calculated on the signed-magnitude images reconstructed with the above algorithm. Mean vessel wall thickness was measured manually on the images obtained with incremental TI and was compared to that from TI*. Finally, a dual-slice rather than a multi-phase version of the sequence (Fig. 1) was tested in four subjects.

## Results

**Figure 3:**
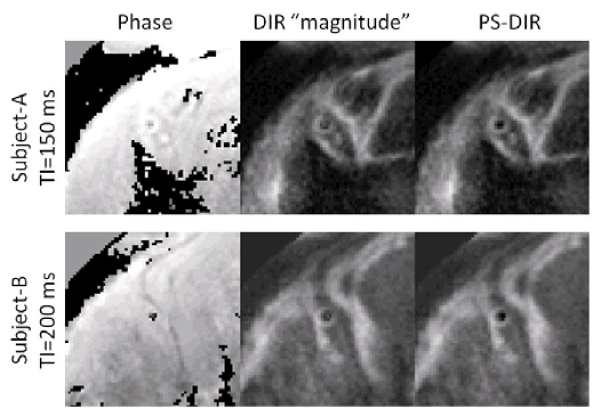
PS-DIR Coronary vessel wall imaging at different TI < TI* in two separate subjects.

**Figure 4:**
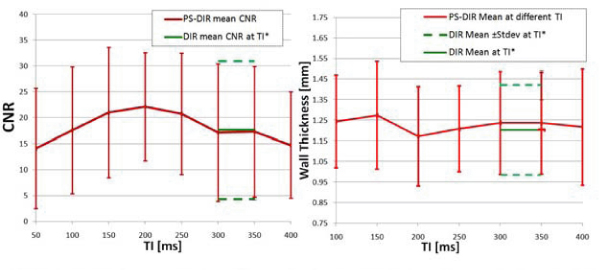
Left: CNR (mean ± stdev) at different TI values in PS-DIR signed images (red) and in DIR magnitude images at TI* (green). Right: Wall thickness at different TI using PS-DIR images (red) and using DIR images at TI*. Note the agreement with the measurements using TI*.

**Figure 5:**
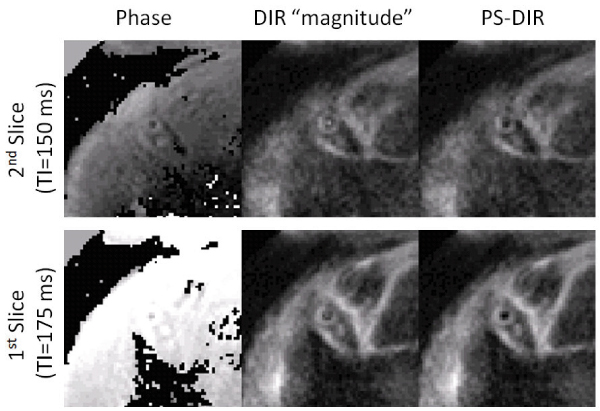
**Dual-slice single breath-hold PS-DIR Coronary vessel wall black-blook imaging**.

Using TI less than TI*, Fig. 3 shows that PS-DIR enables delineation of the coronary artery vessel wall and supports an increased wall-lumen contrast when compared with the conventional DIR in which TI was too short for adequate blood signal-nulling. Consistent with the visual findings, Fig. 4 shows that the CNR significantly increased in PS-DIR over a broad range of TI (150 ms–300 ms). Wall thickness measurements using PS-DIR at different TI values were consistent with those from DIR at TI*. Since the PS-DIR method permits image data collection over a broad range of TI (Fig. 4), multiple slices rather than multiple phases can be obtained at no extra cost in scanning time (Fig 5).

## Discussion

PS-DIR provides a TI-insensitive higher CNR alternative to conventional DIR for coronary vessel wall imaging. TI-insensitivity can be traded for enhanced volumetric coverage at no extra-cost in imaging time.
